# Transdermal Measurement of Glomerular Filtration Rate in Mice

**DOI:** 10.3791/58520

**Published:** 2018-10-21

**Authors:** Lauren Scarfe, Daniel Schock-Kusch, Lorenzo Ressel, Jochen Friedemann, Yury Shulhevich, Patricia Murray, Bettina Wilm, Mark de Caestecker

**Affiliations:** ^1^Division of Nephrology, Department of Medicine, Vanderbilt University Medical Center; ^2^Department of Cellular and Molecular Physiology, University of Liverpool; ^3^MediBeacon GmbH; ^4^Department of Veterinary Pathology and Public Health, Institute of Veterinary Science, University of Liverpool

**Keywords:** Medicine, Issue 140, Glomerular filtration rate, FITC-sinistrin, transdermal, mice, rodents, kidney function

## Abstract

Transdermal analysis of glomerular filtration rate (GFR) is an established technique that is used to assess renal function in mouse and rat models of acute kidney injury and chronic kidney disease. The measurement system consists of a miniaturized fluorescence detector that is directly attached to the skin on the back of conscious, freely moving animals, and measures the excretion kinetics of the exogenous GFR tracer, fluorescein-isothiocyanate (FITC) conjugated sinistrin (an inulin analog). This system has been described in detail in rats. However, because of their smaller size, measurement of transcutaneous GFR in mice presents additional technical challenges. In this paper we therefore provide the first detailed practical guide to the use of transdermal GFR monitors in mice based on the combined experience of three different investigators who have been performing this assay in mice over a number of years.

**Figure:**
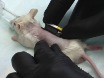


## Introduction

The use of transcutaneous GFR monitors in mice was first reported by Schreiber and colleagues in 2012 and was validated by comparing GFR measurements obtained using this technique, with results obtained by direct measurement of FITC-sinistrin bolus clearance from serial blood samples^1^. To date, there have been 35 peer-reviewed publications in which transcutaneous GFR monitors have been used in rats and mice (a regularly updated list of journal articles and conference abstracts in which the preclinical GFR monitor was used can be found at the MediBeacon website^2^). Transdermal GFR measurements in rats and mice has been described in a number of publications^1^,^3^,^4^,^5^, and a video tutorial demonstrating its use in rats has been published^6^. However, measurement in mice presents additional technical challenges. Here, we provide the first detailed practical guide to the use of transdermal GFR monitors in mice.

There are a variety of reasons why investigators are starting to favor the use of transdermal GFR monitors to assess renal function in rodent models. Transdermal measurement of FITC-sinistrin clearance has been shown to provide a more sensitive and accurate measure of renal function compared to the traditional parameters of renal function such as serum creatinine and blood urea nitrogen (BUN)^7^,^8^. By implementing an improved evaluation algorithm, Friedemann and colleagues demonstrated that the system reaches precision comparable to the gold standard, the constant infusion technique for GFR measurement^3^. Recent studies have also shown that sequential analysis using transcutaneous GFR monitors can be used to study early changes in renal function as well as functional recovery after induction of acute kidney injury (AKI) without interfering with the animals' blood volume or hemodynamics, since the assay does not require sequential blood sampling^9^,^10^. The ability to measure GFR with high precision and sensitivity repeatedly in the same animal makes this technique attractive for a variety of different research disciplines. Transdermal GFR monitors have been used by pharmaceutical companies to assess the toxicity of novel compounds, as well as in universities for basic and translational research.

## Protocol

All animal experiments were performed in accordance with local guidelines in the UK and USA. Experiments conducted at the University of Liverpool were performed under a license granted under the UK Animals (Scientific Procedures) Act 1986 and were approved by the University of Liverpool ethics committee. All animal experiments conducted at Vanderbilt University Medical Center were approved by the Vanderbilt Institutional Animal Care and Use Committee.

### 1. Preparing the FITC-sinistrin

1. Prepare 40 mg/mL FITC-sinistrin in phosphate buffered saline (PBS). NOTE: Aliquots can be stored at -20 °C for several months with no noticeable decrease in quality; however multiple freeze-thaw cycles should be avoided. FITC-sinistrin is light sensitive - keep the tube protected from light.
2. Calculate the volume of FITC-sinistrin required for each mouse:
  1. Weigh each mouse on each day of measurement.
  2. The recommended dose is 0.15 mg FITC-sinistrin per gram body weight.

### 2. Mouse Preparation

1. Prepare separate cages for the mice while undergoing GFR measurements. Provide absorbent paper towels and a few pellets of food.

### 3. Removing Hair from the Mouse (1-2 days before GFR Measurement)

1. Anesthetize the mouse with 3% isoflurane, and once the mouse is asleep, maintain anesthesia with 1.5–2% isoflurane, depending on the breathing rate of the mouse. Place the mouse prone on a heat pad.
2. Use an electric shaver, going against the direction of the fur, to remove most of the fur from one side of the mouse’s back. Shave from the top of the hind legs up to the neck, and across the ribs.
3. Apply a thin layer of depilation cream to the shaved area using a cotton bud (Figure 1A). Move the cotton bud against the direction of the fur to ensure that the cream is applied as close to the skin as possible.
4. Remove the cream after 1–3 min by washing it off with cotton swabs and warm water. Do not perform the measurement if the skin appears very red and irritated after measurement, and do not repeat depilation within 72 h to avoid damaging the skin.

### 4. Preparing the Transdermal GFR Monitor

1. Use one of the two sizes of patches that are available. The first is 2.5 x 3 cm^2^ in size and can be used for measurements in mice directly. The other patches are 6 x 3 cm^2^ in size and are meant to be used in rats or larger animals but can be cut to a smaller size for use in mice.
2. Peel the backing off one side of the patch and stick the GFR device on the adhesive side, positioning the LEDs exactly above the clear window.
3. Cut the excess adhesive patch to fit the size of the battery and stick one side of the patch to the battery.

### 5. Attaching the Transdermal GFR Monitor

1. Anaesthetize the mouse with isoflurane as described in step 3.1 and place the mouse prone on a heat pad. Anesthetize mice only for placement of the transdermal GFR monitor and injection of FITC-sinistrin; allow to recover from anesthesia for the measurement of FITC decay.
2. Clean the pre-shaved skin with 70% ethanol. Place approximately 12 cm of hypoallergenic silk tape under the mouse (Figure 1B; the width of the tape should be reduced to 1.5–2 cm so that it is not too wide for the mouse).
3. Position the tape so that only approximately 2 cm is on the mouse’s right side, and the rest is on the left. Fold over one edge of the right side of tape for easy placement and removal after the measurement. The left-right instructions for steps 5.3 and 5.6 are for device placement on the right side of the animal and can be swapped for device placement on the left side of the animal if required.
4. Connect the battery to the device, remove the backing from the battery and securely place it on top of the device. The device is ready to use and data acquisition starts when the blue light emitting diodes (LEDs) start blinking.
5. Remove the backing from the device and place on to the shaved skin. Position the device such that the window exposing the LEDs is over the ribs – do not have it too close to the spine or limbs (Figure 1C).
6. Secure the device with the white tape. Secure the right side first (Figure 1D), wrapping it tightly around all edges of the device, then wrap the left side around the mouse and device (Figure 1E). Ideally, the left side of the tape only covers the device, and the right side ends under the mouse’s abdomen.
7. Attach the tape by pressing it alongside the circumference of the mouse’s body. The tape needs to be attached firmly, but not tightly. If it is too loose then the device will move around too much and cause movement artefacts. However, it should not be so tight that it restricts breathing or movement or puts too much pressure on the skin.
8. Leave the device untouched for 3 minutes before the FITC-sinistrin injection to allow a steady background reading to be taken. In this time, warm the tail with a heat pad or glove filled with warm water to prepare for tail vein injection (if using this route).

### 6. FITC-sinistrin Injection

1. Prepare an insulin syringe with the calculated amount of FITC-sinistrin required for injection (this can be rounded to the nearest 10 μL).
2. Administer FITC-sinistrin by tail vein or retro-orbital injection. FITC-sinistrin should be administered in one smooth but rapid bolus to avoid multiple peaks on the clearance curve. It is better to administer only a partial dose than to have multiple attempts at administering the FITC-sinistrin.

### 7. Measuring the GFR

1. Place the mouse in a cage on its own to recover from isoflurane anesthesia and for the duration of the measurement period.
2. Observe the mouse in the cage for 1.5 h and then remove the device. Removing the device from the conscious mouse is fast, efficient, and generally well-tolerated by the mouse, but new users may prefer to anaesthetize the mouse for this step.
  1. As one option, anaesthetize the mouse with isoflurane.
  2. As the other option, place the mouse on the wire rack on top of the cage, allowing the mouse to grasp the metal bars whilst the device is removed.
3. Pull off the white plaster tape from underneath the belly in one quick, smooth movement, and remove the device and black plaster from the skin. Be careful that the battery does not disconnect from the device yet.
4. Return the mouse to its home cage.

### 8. Reading and Evaluating the Data

1. Carefully disconnect the battery from the device
2. Connect the device to the USB cable and then connect the cable to the computer
3. Open the reading software (Sensor_ctrl_app.exe)
4. In order, click “connect”, “read”, “re-name”, and “save”, then close the program
5. Process and evaluate data in the analysis software as described in the respective manual

## Representative Results

In this section we present representative results of the use of the transdermal GFR monitor. The transdermal monitor has been used in a variety of mouse strains and models of AKI and CKD^2^.

Figure 2 shows representative FITC-sinistrin clearance curves in male BALB/c mice before and after ischemia reperfusion injury (IRI) with simultaneous contralateral nephrectomy. FITC-sinistrin is rapidly cleared from the circulation in healthy mice (Figure 2A), but clearance is dramatically delayed in mice with AKI (Figure 2B, **C**). In mice with very severe AKI, there may not be any change in FITC-sinistrin fluorescence during the 90-minute measurement period, indicating a complete absence of glomerular filtration (Figure 2C).

Transdermal GFR measurement is minimally invasive and can be used to monitor changes in kidney function in the same mice over multiple time points. Figure 3 depicts changes in GFR determined by sequential transdermal FITC-sinistrin clearance measurements at baseline, and 1, 2 and 4 days after inducing IRI (unilateral ischemia with simultaneous contralateral nephrectomy). Data shown includes FITC-sinistrin clearance half-life (Figure 3A), and GFR (Figure 3B) calculated from the measured FITC-sinistrin clearance half-life, as described by Schreiber *et al*^1^.

In Figure 4, chronic kidney disease (CKD) was induced in male BALB/c mice by performing prolonged unilateral IRI followed by delayed contralateral nephrectomy, as described^11^. GFR was assessed by transdermal FITC-sinistrin clearance on day 26 after the initial IRI. The increase in FITC-sinistrin half-life (Figure 4A), and therefore the decrease in GFR (Figure 4B), indicates impaired renal function in these mice. These data demonstrate that transcutaneous GFR measurement can be used to measure changes in renal function in mice with CKD.

Figure 5A shows that FITC-sinistrin half-life correlates closely with semi-quantitative histological assessment of tubular injury over the full range of GFR measurements in uninjured mice and in mice with different severities of IRI-induced AKI. In contrast, serum creatinine and blood urea nitrogen (BUN) showed a positive but weaker correlation with FITC-sinistrin clearance (Figure 5B**, C**), indicating that transcutaneous GFR measurements provide a more reliable measure of renal injury (tubular injury scores) following IRI-induced AKI than either serum creatinine or BUN.

**Figure:**
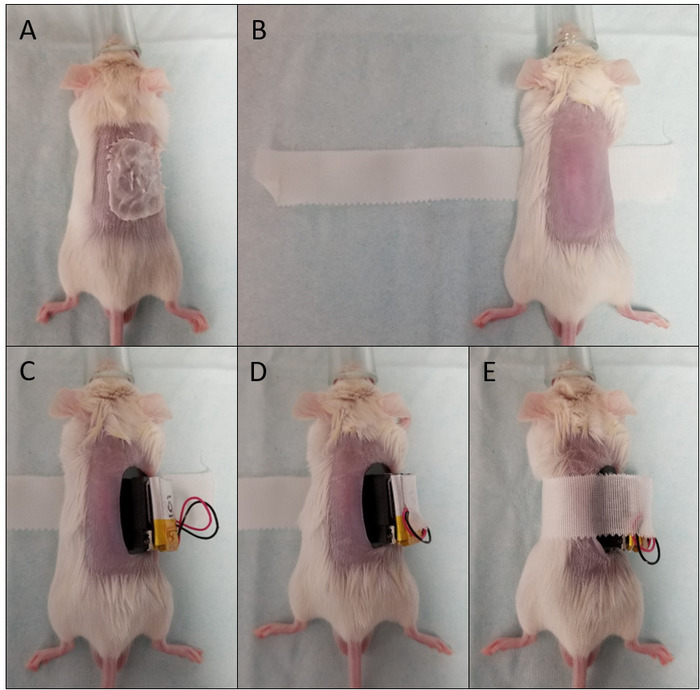

**Figure 1: Attaching the transdermal GFR monitor. **Photographs of hair removal (**A**), placement of the tape under the mouse (**B**), placement of the device on the mouse's skin (**C**), and securing the device by wrapping the tape around the mouse and device (**D-E**) Please click here to view a larger version of this figure.

**Figure:**
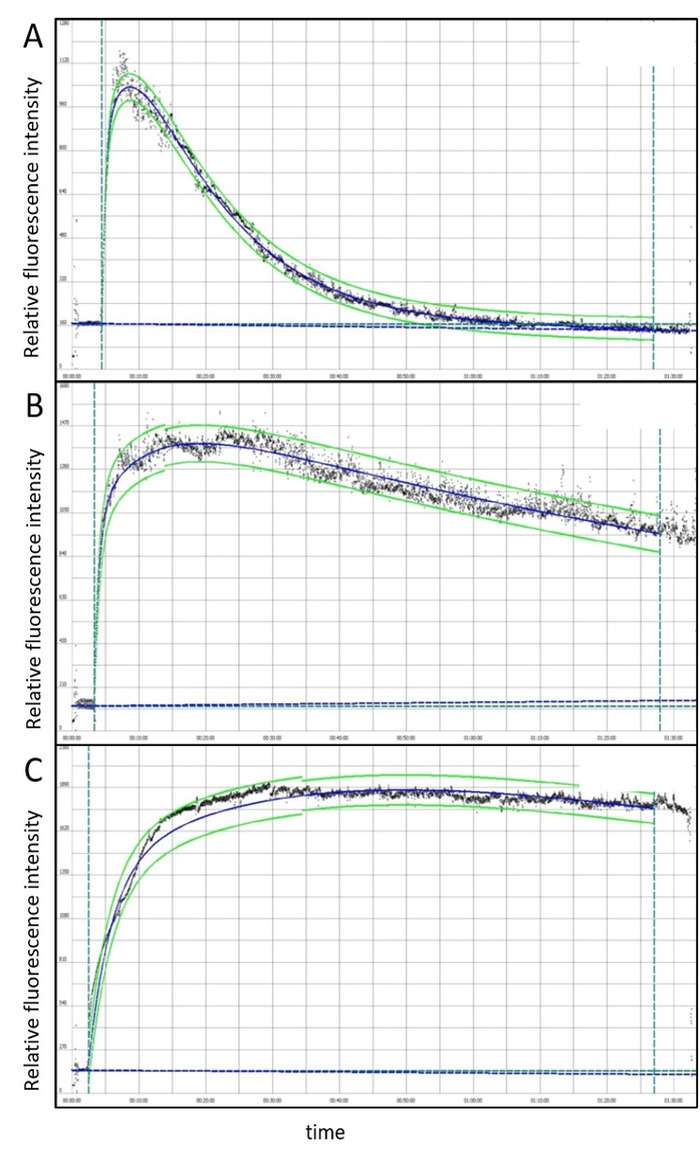

**Figure 2: Example FITC-sinistrin clearance curves in male BALB/c mice before and after ischemia reperfusion injury (IRI) with simultaneous contralateral nephrectomy.** Clearance curves at baseline (**A**), and one day after IRI surgery (**B**) in the same mouse, indicating impaired renal function in this mouse. (**C**) Clearance curve from a more severely injured mouse one day after IRI surgery. There was no clearance of FITC-sinistrin during the measurement period, indicating renal failure. Black data points represent raw data, blue lines represent the 3-compartment fit, and green lines represent 95% confidence intervals. Please click here to view a larger version of this figure.

**Figure:**
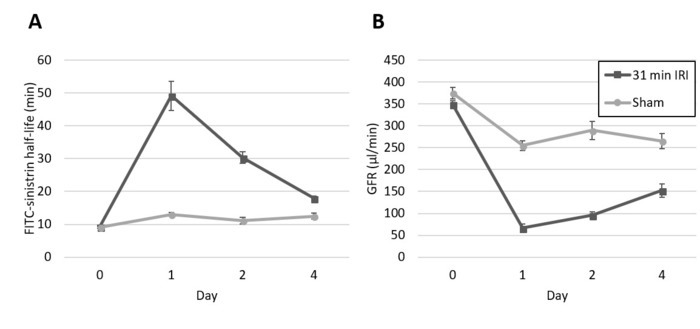

**Figure 3: Male BALB/c mice, age 8-10 weeks underwent unilateral ischemia with simultaneous contralateral nephrectomy (n=5).** GFR was assessed at baseline and on days 1, 2, and 4 after surgery, and compared with sham-operated control mice (n=5). FITC-sinistrin half-life in (**A**) was used, along with the body weight of the mice, to calculate GFR in (**B**). Data points represent individual animals, and error bars represent mean and standard error. Please click here to view a larger version of this figure.

**Figure:**
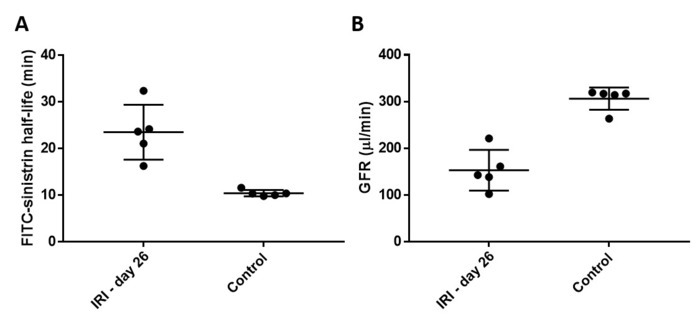

**Figure 4: Male BALB/c mice age 8-10 weeks underwent unilateral ischemia with delayed contralateral nephrectomy at day 8 (n=5).** GFR was assessed by on day 26 and was compared to age-matched healthy control mice (n=5). FITC-sinistrin half-life in (**A**) was used, along with the body weight of the mice, to calculate GFR in (**B**). Data points represent individual animals, and error bars represent mean and standard deviation. Tubular injury was scored 0-50 based on the degree of necrosis and cast formation by a blinded observer (L.R.) on Periodic acid-Schiff-stained kidney sections. This method was adapted from Wang and colleagues^12^. Please click here to view a larger version of this figure.

**Figure:**
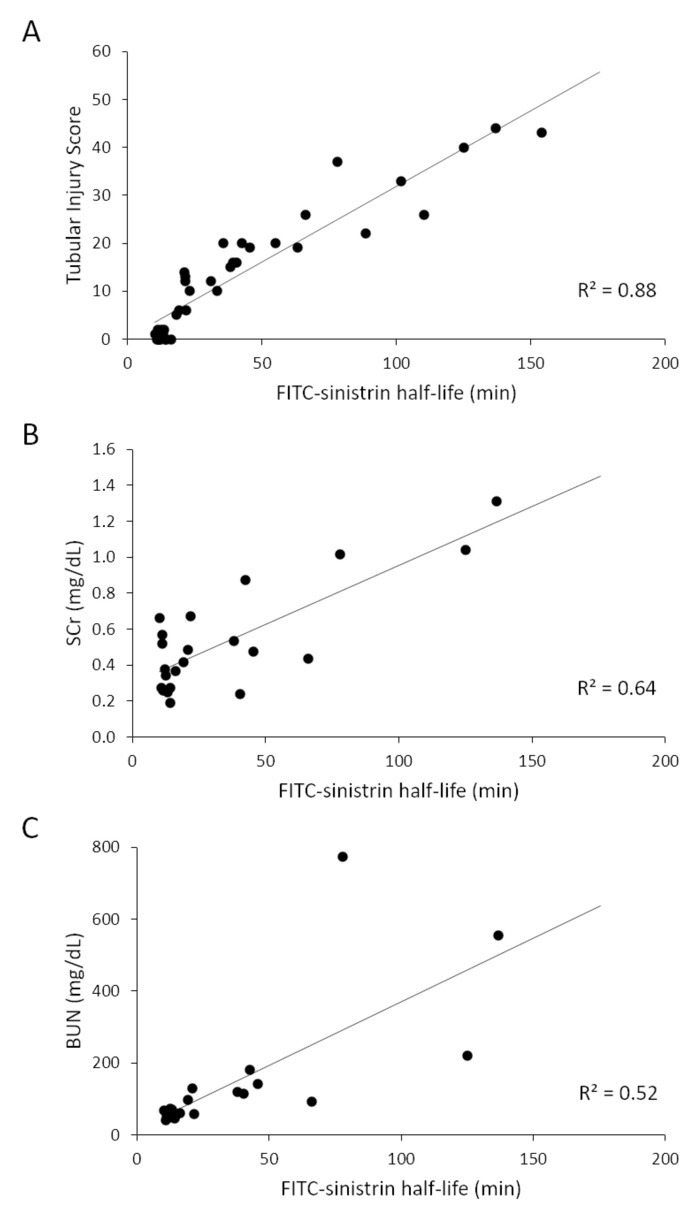

**Figure 5: Correlation of three measures of kidney function/damage (histological evaluation of tubular injury (n=39), serum creatinine (n=30) and blood urea nitrogen (BUN) (n=30)) with FITC-sinistrin clearance (half-life).** Male BALB/c mice underwent varying periods of unilateral renal pedicle clamping (25–45 min) or sham surgery, with simultaneous contralateral nephrectomy to induce different severity of AKI, and renal function parameters and histopathology were assessed at day 4 after IRI. The tubular injury score showed a strong positive correlation with FITC-sinistrin clearance (**A**; R^2^ = 0.88), whereas serum creatinine (**B**), and BUN (**C**) both showed positive but weaker correlation with FITC-sinistrin clearance (R^2^ = 0.64 and 0.52, respectively). Please click here to view a larger version of this figure.

## Discussion

This manuscript and the accompanying training video provide practical guidelines for the use of transdermal GFR monitors in mice. The most critical steps in the procedure are the correct attachment of the device on the animal's back, and securely wrapping the tape around the abdomen. The best position is either slightly left or right of the midline, over the ribcage. The patch and device need to be firmly attached to the skin, but they should not be so tight that they restrict breathing, movement, or affect skin blood circulation under the device, as this would lead to faulty/inaccurate measurements. In addition, since monitoring occurs in conscious mice after they have recovered from anesthesia, correct placement of the device on the part of the body with lowest interference from movement results in transdermal measurements with little movement artefacts. For this reason, it is important that the device is not placed too close to the upper limbs so that the mice can move their shoulders freely.

It is important to depilate the mice one to two days before GFR measurement, as depilation affects the measurement of FITC-sinistrin clearance, with preliminary data indicating that depilation immediately before measurement of transdermal GFR increases the apparent half-life of FITC-sinistrin. The mechanism for this is unknown. Therefore, in order to obtain reliable measurements over multiple time points and between experiments, it is advisable to depilate the mice in advance, to allow the skin to recover from this process before proceeding with GFR measurements. Depilation cream should not be applied to the same area of skin within 72 h of a prior application, to avoid chemical damage to the skin. In many cases, the fur re-growth takes several days or up to a week, and so re-application of depilation cream within 72 h can easily be avoided. 

Because up to 50% of serum creatinine is excreted by tubular section in mice^13^, and because there is increased reabsorption of urea from renal tubules when mice are dehydrated^14^, serum creatinine and BUN are poor markers of renal function. However, because of their convenience, these assays continue to be used as the main measure of renal function in preclinical studies of AKI and CKD in mice. However, consistent with major contribution of tubular secretion to creatinine excretion in mice with normal or near normal renal function^13^, serum creatinine showed little correlation with FITC-sinistrin clearance at high clearance rates (low FITC-sinistrin half-life), indicating that creatinine is an insensitive measure of renal function in mice with mild kidney injury. In contrast, while BUN correlates well with FITC-sinistrin clearance in mice with mild renal impairment, there is poor correlation between BUN and FITC-sinistrin clearance in mice with more severe kidney injury (high FITC-sinistrin half-life). This is likely caused by effects of urea reabsorption associated with dehydration in sick animals with severe kidney injury.

A major advantage of the transdermal GFR measurement, compared to all other bolus clearance or constant infusion techniques for GFR measurement, is that it does not require carefully timed blood or urine collections. These can be particularly challenging in mice as they have low total blood volumes and urinary output as compared with rats. Moreover, mice need to be handled only for attachment of the device and injection, but not for multiple venipunctures, as required for classical bolus clearance experiments^15^. Furthermore, the duration of anesthesia is short, and as such it is possible to perform repeated measurements in individual mice over time. The frequency at which measurements can be performed primarily depends on the health status of the mice, the researcher’s aptitude for intravenous injections, and local institutional regulations on repeated anesthesia sessions. In healthy, uninjured mice, transdermal GFR measurements can be performed daily, with minimal or no adverse effects on the mouse. However, injured mice suffering from AKI or CKD are unlikely to tolerate repeated anesthesia sessions as well as the healthy mice, and thus the frequency of measurements should be reduced. 

The main limitation of transdermal GFR measurement, as compared with bolus clearance methods to measure GFR in mice is that the excretion kinetics are only measured as change in relative fluorescence intensity over time, and not as absolute tracer concentrations. Because of this, it is only possible to measure the rate constant of the single exponential decay of the excretion kinetic, which is a very close estimate of GFR normalized on extracellular volume^16^. To express GFR in mL/min, the extracellular volume of the animal has to be estimated using a conversion factor that was established in prior studies in which simultaneous measurements of plasma concentrations of FITC-sinistrin were performed^1^. However, this conversion factor may not correctly estimate extracellular fluid volumes equally well in all mice, since fluid volume may be affected by a variety of extraneous factors including age, sex, hydration status (which may be affected by surgical interventions as well as kidney injury), and weight^17^. However, unlike the bolus dosing method to assess GFR in mice, transcutaneous GFR measurement is subject to less operator-dependent variability as it is not affected by dosing errors or by errors in timing of blood collections.

Another limitation of the transcutaneous GFR measurement technique is that baseline signal shifts may occur during the course of the measurement due to bleaching of skin fluorophores and the anesthesia required for device attachment and tracer injection. This limitation was addressed by Friedemann and colleagues by implementing a correction algorithm^3^. The implementation of this algorithm led to an improvement in precision of the transdermal technique comparable to a constant infusion technique of GFR assessment.

A frequently asked question is whether skin pigmentation in different mouse strains affects the transdermal FITC-sinistrin clearance. Skin pigmentation reduces the FITC-sinistrin signal intensity since dark pigments absorbs the blue excitation and the green emission signals from FITC-sinistrin measurements. However, the excretion rate of FITC-sinistrin is independent of the overall signal intensity. Furthermore, while the measured signal is lower, the background signal is also lower in pigmented mice. Because the background signal is a mixture of autofluorescence of skin fluorophores and reflection of the excitation light, we have found that the background-to-maximum signal ratio is comparable, or even improved, in pigmented animals. In addition, movement artifacts, which are caused by exposure of the surrounding skin to reflected light, are reduced in pigmented mice since the reflected light is also absorbed by pigmented skin.

In conclusion, the technique we have presented allows precise measurement of GFR in conscious, freely moving mice of all skin types. As the technique is independent of blood sampling, it can be used repeatedly on the same animal for longitudinal observations in CKD models, as well as for the measurement of rapid changes of GFR that occur after induction of AKI.

## Disclosures

D S-K, JF and YS are employees at MediBeacon GmbH the manufacturer and distributor of the transdermal GFR monitor. D S-K and JF are inventors on patents and patent applications for the presented technology.
